# Circulating tumor cells in hepatocellular carcinoma: a pilot study of detection, enumeration, and next-generation sequencing in cases and controls

**DOI:** 10.1186/s12885-015-1195-z

**Published:** 2015-03-31

**Authors:** Robin K Kelley, Mark Jesus M Magbanua, Timothy M Butler, Eric A Collisson, Jimmy Hwang, Nikoletta Sidiropoulos, Kimberley Evason, Ryan M McWhirter, Bilal Hameed, Elizabeth M Wayne, Francis Y Yao, Alan P Venook, John W Park

**Affiliations:** 1 Helen Diller Family Comprehensive Cancer Center and The Liver Center, University of California San Francisco (UCSF), 550 16th St., Box 3211, San Francisco, CA 94143 USA; 2 Helen Diller Family Comprehensive Cancer Center, UCSF, San Francisco, CA 94143 USA; 3 Department of Molecular and Medical Genetics, Oregon Health Sciences University, 3181 SW Sam Jackson Park Road, Mail Code #L103, Portland, OR 97239 USA; 4 University of Vermont Medical Center, 89 Beaumont Ave., Burlington, VT 05405 USA; 5 Department of Pathology, UCSF, 513 Parnassus Ave., San Francisco, CA 94143 USA; 6 Division of Hepatology and Liver Transplant, UCSF, 513 Parnassus Ave., S-357, San Francisco, CA 94143 USA; 7 Department of Transplantation-Abdominal, UCSF, 513 Parnassus Ave., S-357, San Francisco, CA 94143 USA; 8 Division of Hepatology and Liver Transplant and The Liver Center, UCSF, 513 Parnassus Ave., S-357, San Francisco, CA 94143 USA

**Keywords:** Hepatocellular carcinoma (HCC), Circulating tumor cells (CTC), EpCAM, Sequencing

## Abstract

**Background:**

Circulating biomarkers are urgently needed in hepatocellular carcinoma (HCC). The aims of this study were to determine the feasibility of detecting and isolating circulating tumor cells (CTCs) in HCC patients using enrichment for epithelial cell adhesion molecule (EpCAM) expression, to examine their prognostic value, and to explore CTC-based DNA sequencing in metastatic HCC patients compared to a control cohort with non-malignant liver diseases (NMLD).

**Methods:**

Whole blood was obtained from patients with metastatic HCC or NMLD. CTCs were enumerated by CellSearch then purified by immunomagnetic EpCAM enrichment and fluorescence-activated cell sorting. Targeted ion semiconductor sequencing was performed on whole genome-amplified DNA from CTCs, tumor specimens, and peripheral blood mononuclear cells (PBMC) when available.

**Results:**

Twenty HCC and 10 NMLD patients enrolled. CTCs ≥ 2/7.5 mL were detected in 7/20 (35%, 95% confidence interval: 12%, 60%) HCC and 0/9 eligible NMLD (*p* = 0.04). CTCs ≥ 1/7.5 mL was associated with alpha-fetoprotein ≥ 400 ng/mL (*p* = 0.008) and vascular invasion (*p* = 0.009). Sequencing of CTC DNA identified characteristic HCC mutations. The proportion with ≥ 100x coverage depth was lower in CTCs (43%) than tumor or PBMC (87%) (*p* < 0.025). Low frequency variants were higher in CTCs (*p* < 0.001).

**Conclusions:**

CTCs are detectable by EpCAM enrichment in metastatic HCC, without confounding false positive background from NMLD. CTC detection was associated with poor prognostic factors. Sequencing of CTC DNA identified known HCC mutations but more low-frequency variants and lower coverage depth than FFPE or PBMC.

**Electronic supplementary material:**

The online version of this article (doi:10.1186/s12885-015-1195-z) contains supplementary material, which is available to authorized users.

## Background

Hepatocellular carcinoma (HCC) is a grim, heterogeneous disease with limited treatment options despite its enormous global impact as the third leading cause of cancer death worldwide [1]. Conventional liver imaging modalities for diagnosis and staging are imprecise and can result in underestimation of the true extent of disease, with microvascular invasion and multifocal tumors often identified incidentally at resection or transplant and associated with significantly poorer prognosis [2,3]. Translational research efforts to better understand the complex tumor biology of HCC, define biomarkers, and identify novel therapeutic targets are further limited by a scarcity of annotated, untreated tumor specimens, owing to the acceptance of radiographic diagnosis without tissue confirmation, the prevalence of liver-directed therapy before transplantation, and the risks associated with tumor biopsy in this population [4,5]. Non-invasive biomarkers for diagnosis and molecular characterization are urgently needed to overcome these pervasive challenges in HCC.

Circulating tumor cells (CTCs) in the peripheral blood are a biomarker of poor prognosis in multiple epithelial tumor types [6,7]. The CellSearch System (Veridex LLC, Raritan, New Jersey, U.S.A) is an FDA-cleared device for CTC detection using enrichment for cells in the blood expressing the epithelial cell adhesion marker (EpCAM) [6]. The absolute numbers of CTCs detected and changes on therapy have been associated with survival and treatment response in breast, colon, and prostate cancers [8-13]. Multiple small studies have examined CTCs in patients with HCC using EpCAM- and non-EpCAM-based enrichment methods, with detection rates ranging from approximately 30% to over 80% depending on methodology and population [14-17]. As in other epithelial tumor types, the detection of CTCs by CellSearch correlates with poor prognosis in HCC cohorts, including increased recurrence risk after resection and shorter overall survival [14,15].

In order to study CTCs as a biomarker in HCC, however, it is essential to establish that circulating epithelial cells in HCC populations are true tumor cells, rather than benign epithelial cells released into circulation as a consequence of the underlying inflammation or aberrant vasculature associated with liver disease. Though the detection of CTCs by CellSearch is extremely rare in healthy volunteers or patients with benign conditions [6,10], there is limited data describing the incidence of circulating EpCAM-positive epithelial cells in the context of cirrhosis, viral hepatitis, or other causes of liver injury, conditions present in the majority of patients with HCC [14].

Beyond detection and enumeration, isolation of CTCs in cancer patients holds great promise as a “liquid biopsy”, a non-invasive means of accessing real-time tumor tissue in the metastatic state for molecular profiling. Array comparative genomic hybridization has demonstrated concordance of characteristic copy number aberrations between CTC-derived DNA and archival primary tumor samples in breast, colon, prostate, and lung cancer [12,18-20]. Next-generation sequencing technologies now have the ability to sequence very small amounts of input DNA with high accuracy [21,22]. Illumina MiSeq technology can detect characteristic driver mutations in single CTCs derived from patients with metastatic colorectal cancer, concordant with the mutational profile of paired primary tumor specimens [18]. To date, the feasibility of efficient CTC isolation and molecular profiling, e.g. next-generation DNA sequencing, has not been reported in HCC.

We conducted this study to determine the proportion of metastatic HCC patients with detectable circulating EpCAM-positive epithelial cells using the CellSearch System, compared to a relevant control cohort of patients with liver disease, hypothesizing that circulating EpCAM-positive cells are actual tumor cells rather than benign epithelial cells. To characterize their prognostic significance, CTC levels were examined for association with clinical covariates including alpha-fetoprotein (AFP) levels, the presence of vascular invasion, and overall survival. To explore the potential for CTCs to serve as a source of tumor DNA for genomic profiling in HCC, next-generation sequencing using a targeted cancer gene panel was performed using whole genome-amplified DNA derived from pooled purified CTCs, along with DNA from paired archival, paraffin-embedded tumor tissue and peripheral blood mononuclear cells when available.

## Methods

### Study design

This pilot study was a non-therapeutic, minimally-invasive biomarker study. The trial was approved by the UCSF Committee on Human Research. All patients provided written informed consent for specimen collection and genetic testing of tumor and germline DNA. The study was conducted in accordance with the Declaration of Helsinki and Good Clinical Practice.

The primary endpoint was incidence of CTCs detected in metastatic HCC patients compared to a control cohort with NMLD. Secondary endpoints were enumeration of CTCs in each cohort, association with clinical and pathologic characteristics including alpha fetoprotein (AFP) level, tumor vascular invasion, and etiology of liver disease in the HCC cohort, and association with overall survival in the HCC cohort. An exploratory endpoint was to describe performance of and somatic mutations identified by next-generation sequencing of CTC whole-genome-amplified DNA along with paired tumor and germline DNA when available.

### Patient selection

HCC patients were recruited at the UCSF Helen Diller Family Comprehensive Cancer Center. Principal inclusion criteria were: radiographic [4] or histologic diagnosis of American Joint Committee on Cancer (AJCC) stage IV HCC; ≥ 6 weeks post biopsy, surgery, liver-directed interventions, or other invasive procedures; no prior systemic therapy or ≥ 4 weeks since last dose of sorafenib or other systemic therapy for advanced HCC. Non-malignant liver disease (NMLD) control cohort patients were recruited at the UCSF Gastroenterology and Liver Disease Clinic. Principal inclusion criteria were: diagnosis of active hepatitis of any etiology plus clinical or pathologic diagnosis of cirrhosis or hepatic fibrosis (any stage); no evidence liver tumor on ultrasound or cross-sectional imaging within 6 months; AFP ≤ 20 ng/mL within 6 months; ≥ 6 weeks post biopsy, surgery, or other invasive procedures; no prior history of HCC.

### Specimen collection

Approximately 30 mL of whole blood was obtained from study subjects at a single time-point. For HCC patients with available archival tumor tissue from prior biopsy or resection, approximately five 10-micron sections of formalin-fixed, paraffin-embedded (FFPE) tumor along with a matching H&E slide were collected from the pathology files of the University of California, San Francisco. Banked frozen aliquots of peripheral blood mononuclear cell (PBMC) were obtained when available from HCC cohort patients.

### Circulating tumor cell enumeration

CTCs were isolated from 7.5 mL whole blood and enumerated using the CellSearch System (Veridex LLC, Raritan, NJ) [6-8]. Briefly, specific antibodies to EpCAM were used to enrich for epithelial cells. A mixture of fluorescently-labeled monoclonal antibodies to cytokeratin and the nuclear dye DAPI were used to select for nucleated, keratin-positive cells. CTCs were defined as nucleated, EpCAM-positive cells that stain positive for cytokeratin and negative for leukocyte common antigen, CD45 [6]. Labeled cells were enumerated using semi-automated fluorescence-based microscopy. Analysis was performed by a trained technician blinded to diagnosis (HCC versus NMLD).

### Immunoenrichment and fluorescence-activated cell sorting (IE/FACS)

A novel EpCAM-based immunoenrichment (IE)/fluorescence-activated cell sorting (FACS) procedure has been developed to isolate purified CTCs without contamination from normal blood cells and has demonstrated correlation with CellSearch System CTC enumeration [12,19,23]. For patients found to have > 10 CTCs in 7.5 mL of whole blood by CellSearch System, IE/FACS was then performed to isolate purified CTCs as has been previously described [12,24]. Briefly, approximately 15–20 mL of whole blood was incubated with immunomagnetic particles coated with two different monoclonal antibodies to EpCAM, one conjugated to magnetic particles and the other to a fluorophore. FACS was used to isolate nucleated, EpCAM-positive, CD45-negative cells.

### Whole genome amplification (WGA)

A ligation-adaptor method of WGA was performed on whole cell lysates from pooled CTCs isolated by IE/FACS using a GenomePlex whole genome amplification kit (WGA4, Sigma-Aldrich) according to the manufacturer’s instructions [12,25]. DNA was randomly fragmented and converted to polymerase chain reaction (PCR)-amplifiable library molecules flanked by universal priming sites. PCR amplification of library molecules was performed using universal oligonucleotide primers.

### DNA extraction from tumor tissue and peripheral blood mononuclear cells (PBMC)

Tumor-containing FFPE sections were identified and marked by a hepatopathologist (KE). DNA was extracted from FFPE sections as well as from banked PBMC using QIAmp kits (Qiagen) according to the manufacturer’s instructions. DNA concentration was quantified using PicoGreen.

### Ion semiconductor NGS

Sequencing of DNA extracted from CTCs, FFPE, and PBMC was performed by TMB in the Spellman Laboratory at Oregon Health Sciences University. From each sample, 10 ng DNA was PCR-amplified using AmpliSeq Cancer Panel Primer Pools and Library Kit 2.0 to generate 190 multiplexed amplicons (representing 46 cancer-related genes) [21]. Up to 11 barcoded samples were multiplexed on Ion 318 chips. Sequencing was performed on a Personal Genome Machine (PGM) sequencer (Ion Torrent) using the Ion PGM 200 sequencing kit. Torrent Suite software version 4.0.1 was employed to analyze read counts and quality. Variant Caller software version 4.0.1 identified variants. Coverage Analysis software version 4.0.1 determined target coverage. To minimize false positives, variants were required to have sequencing depth of at least 20x, an allele frequency of 5 percent, and not be present in any of the 3 PBMC samples sequenced. Variant calls were filtered against the Single Nucleotide Polymorphism Database (dbSNP) version 132, using the software ANNOVAR. Protein-altering variants were predicted by Mutation Assessor version 2 (http://mutationassessor.org).

### Statistical analysis

Based upon the a priori hypothesis that approximately 50% of the HCC cohort and none of the NMLD cohort would have detectable CTCs by CellSearch, the planned sample size for this pilot study was 20 patients with metastatic HCC and 10 patients with NMLD, to permit estimation of proportion of detectable CTCs with 95% confidence intervals (CI) as (0.30, 0.70) in the HCC cohort and (0.01, 0.26) in the NMLD cohort. The incidence and number of detectable CTCs were analyzed using frequency and proportions with 95% CI and compared between HCC and NMLD cohorts using the Wilcoxon-Kruskal-Wallis rank test. Cut-points of ≥ 1, ≥ 2, ≥ 3, and ≥ 5 CTCs/7.5 mL were examined based upon published literature in HCC and other tumor types [8,10,14,15]. Wilcoxon-Kruskal-Wallis rank testing was also used to determine association between the presence of detectable CTCs by CellSearch System, AFP elevation using ≥ 400 ng/mL as an established prognostic cut-point [26,27], and the presence of vascular invasion (all binary variables). In the HCC cohort, overall survival was measured in months from date of CTC blood draw to the date of death with censoring at date of last known vital status if lost to follow-up. Kaplan-Meier methods were used to determine the impact of CTCs at each cut-point and conventional prognostic factors on overall survival. The CTC level, AFP value of 400 ng/mL, and presence of macrovessel invasion were used to dichotomize for univariate analyses. The Child Pugh score and etiology of liver disease were also examined. A *p* value of < 0.05 was considered statistically-significant under log-rank tests. Sequencing coverage depth was compared between sample types using two-tailed t-tests assuming unequal variance. Variant calls were reported descriptively due to small sample size.

## Results

### Patient characteristics

**Figure 1:**
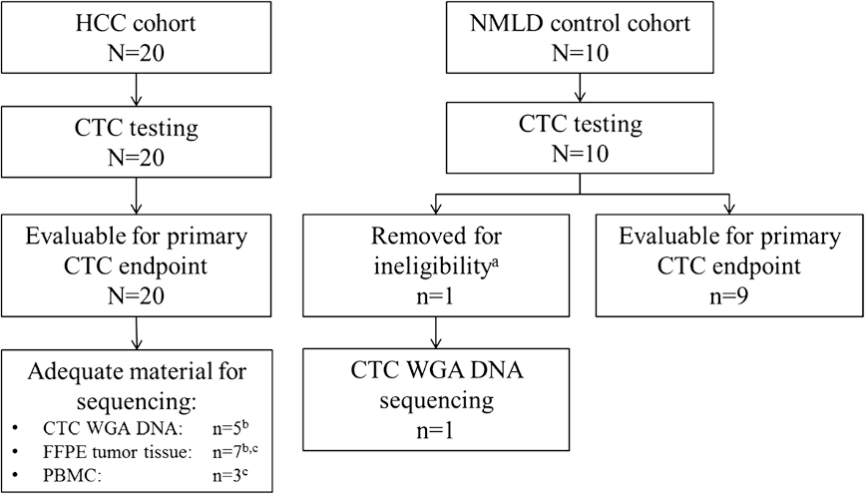
**Study subject enrollment and samples tested.**^a^One patient enrolled to NMLD control cohort was removed for ineligibility due to new finding of liver mass with portal vein thrombosis on imaging after enrollment. CTC testing in this patient showed 20 CTCs per 7.5 mL peripheral blood. ^b^One sample each of CTC and FFPE did not yield sufficient DNA for sequencing. ^c^4 primary and 3 metastatic tumor FFPE samples were available from 7 of the HCC cohort cases. Paired CTC WGA DNA and FFPE tumor tissue were available in 2 cases, one of which also had PBMC available. Paired FFPE tumor tissue and PBMC were available from 2 additional cases.

Twenty patients with a diagnosis of metastatic HCC (HCC cohort) and 10 patients with underlying non-malignant liver disease without cancer (NMLD cohort) were prospectively enrolled between June 2011 and April 2012. All HCC patients were followed to date of death. Baseline patient characteristics are shown in Table 1. The median overall survival in the HCC cohort was 9.44 months from date of CTC blood draw. One NMLD cohort patient with HCV cirrhosis (Hep 25) was found to have a liver mass with adjacent portal vein thrombosis on a surveillance ultrasound after enrollment and was excluded based upon a suspected new diagnosis of HCC, resulting in 9 eligible patients in the NMLD cohort. The patient was subsequently lost to follow up. Figure 1 displays the study subject enrollment and samples tested.

**Table 1:** **Patient characteristics**

	HCC cohort (*n* = 20)	NMLD control Cohort (*n* = 10)
Median age (range) (years)	61.5 (50–82)	26-91 (53.5)
Male/female (*n*)	20/0	9/1
Etiology of liver disease (%)		
HBV	25	20
HCV	45	60
Co-infection HBV + HCV^a^	10	0
ETOH	5	10
NAFLD	10	0
PSC	0	10
Unknown	5	0
Race/ethnicity (%)		
African-American	5	10
Asian	35	10
Caucasian	55	70
Hispanic/Latino	5	30
Non-Hispanic/Latino	50	40
Native American	5	0
Other/unknown	0	10
Child Pugh score (%)		
A/B/C/unknown	70/25/5/0	30/30/30/10
Median AFP (range) (ng/mL)	492 (3.8-587,134)	5.5 (1.7-17.2)
BCLC score C (%)^b^	100	N/A
Vascular invasion (%)	65	N/A
Extrahepatic spread (%)^b^	100	N/A
Median overall survival (months)	9.4 months	Not measured

Key: HBV = hepatitis B virus. HCV = hepatitis C virus. ETOH = alcohol. NAFLD = non-alcoholic fatty liver disease. PSC = primary sclerosing cholangitis. BCLC = Barcelona Clinic Liver Cancer. N/A = not applicable.

^a^Defined as HCV antibody positive plus either HBV surface antigen and/or core antibody positive.

^b^BCLC C and presence of extrahepatic spread were required eligibility criteria for HCC cohort.

### CTC detection and enumeration by CellSearch

**Figure 2:**
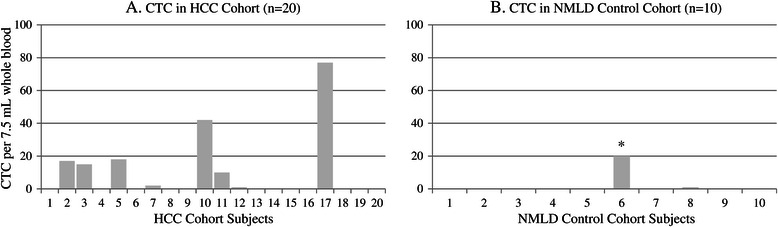
**CTC detection and enumeration by CellSearch.** Figure 2 depicts the CTC count per 7.5 mL whole blood by CellSearch in the HCC cohort **(A)** and NMLD control cohort **(B)**. *One patient in NMLD cohort who was removed for ineligibility due to new liver mass with portal vein thrombosis was found to have 20 CTCs per 7.5 mL peripheral blood.

Figure 2 depicts the number of CTCs detected in each patient. At least 1 CTC per 7.5 mL was detected in 8 of 20 (40%, 95% CI: 17%, 64%) HCC patients and 1 of 9 (11%, 95% CI: 0, 37%) eligible NMLD patients (*p* = 0.1, Wilcoxon-Kruskal-Wallis rank test). At least 2 CTC per 7.5 mL were detected in 7 of 20 (35%, 95% CI: 12%, 60%) HCC patients and 0 of 9 eligible NMLD patients (*p* = 0.04, Wilcoxon-Kruskal-Wallis rank test). Among the HCC cohort patients, at least 1 CTC per 7.5 mL was detected in 7 of 10 (70%, 95% CI: 35%, 100%) with AFP ≥ 400 ng/mL, versus 1 of 10 (10%, 95% CI: 0, 33%) with AFP < 400 ng/mL (*p* = 0.008). At least 1 CTC per 7.5 mL was detected in 8 of 13 (62%, 95% CI: 31%, 92%) with vascular invasion versus 0 of 7 without (*p* = 0.009) (Wilcoxon-Kruskal-Wallis rank tests). The NMLD control cohort patient Hep 25 who was removed for ineligibility (due to new liver mass with thrombosis consistent with HCC) was found to have 20 CTCs per 7.5 mL peripheral blood. Another NMLD cohort patient with alcoholic cirrhosis had 1 CTC detected per 7.5 mL peripheral blood. It is noteworthy that the single eligible NMLD control patient with detectable CTCs (1 in 7.5 mL) subsequently developed new infiltrative changes in the liver on a surveillance ultrasound, raising the possibility of underlying tumor though no formal HCC diagnosis was made before his death of complications of cirrhosis approximately 13 months after CTC blood draw.

**Figure 3:**
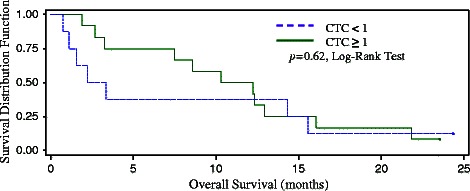
**Kaplan-Meier survival curve in HCC cohort by CTC strata.** Overall survival was measured from date of CTC blood draw to date of death. The median overall survival was 2.8 months in patients with CTC ≥ 1/7.5 mL (95% CI: 1.08, 15.5) versus 11.3 months in patients with CTC < 1/7.5 mL (95% CI: 7.49, 12.9) though the difference was not statistically significant in this small sample (*p* = 0.62, Log-Rank test).

The median overall survival (OS) in the HCC cohort was 9.4 months. Among HCC cohort patients with at least 1 CTC per 7.5 mL, the median OS was 2.8 months (95% CI: 1.08, 15.5), versus 11.3 months (95% CI: 7.49, 12.9) for those without CTCs detected, although the difference was not statistically significant (*p* = 0.62, Log-Rank test) (Figure 3). In univariate analysis of CTC levels and conventional prognostic factors (Table 2), none showed significant effect on overall survival, though analyses were limited by small sample sizes; no further multivariate analysis was performed.

**Table 2:** **Univariate analysis of CTC levels and conventional prognostic factors with overall survival**

HCC Cohort (*n* = 20)	Mean overall survival (months) (standard error)	Median overall survival (months) (95% CI)	*p*value (Log-Rank test)
CTC per 7.5 mL			
< 1.0 (*n* = 12)	10.96 (1.95)	11.29 (2,69, 16.06)	
1.0 (*n* = 8)	8.49 (3.63)	2.76 (0.72, 15.54)	0.6179
<2.0 (*n* = 13)	10.37 (1.89)	10.32 (3.25, 12.91)	0.8021
≥2.0 (*n* = 7)	9.23 (4.11)	2.20 (0.72, 15.54)	0.8510
<3.0 (*n* = 14)	9.74 (1.86)	9.45 (2.69, 12.91)	
≥3.0 (*n* = 6)	10.50 (4.62)	8.26 (0.72, 29.14)	
Median AFP (ng/mL)			
<400 (*n* = 10)	11.20 (2.29)	11.32 (2.69, 16.07)	0.4058
≥400 (*n* = 10)	8.73 (2.92)	5.39 (0.72, 14.32)	
Macrovessel invasion			
No (*n* = 7)	10.12 (2.48)	10.32 (2.69, 12.91)	0.7493
Yes (*n* = 13)	10.45 (2.82)	8.58 (1.58, 15.54)	
Child Pugh score (%)			
A (*n* = 14)	10.69 (1.87)	11.32 (2.20, 15.54)	
B (*n* = 5)	9.29 (5.39)	3.25 (0.72, 29.14)	0.7181
C (*n* = 1)	I		
Etiology of liver disease (%)			
HBV (*n* = 5)	10.28 (3.83)	8.58 (2.20, 21.85)	
HCV (*n* = 9)	10.41 (1.96)	12.62 (1.91, 15.54)	0.9324
HBV + HCV (*n* = 2)	I	I	
ETOH (*n* = 1)	I	I	
NAFLD (*n* = 2)	I	I	
Unknown (*n* = 1)	I	I	

Kaplan-Meier methods were used to determine the impact of CTC at each cut-point and conventional prognostic factors on overall survival. The CTC level, AFP value of 400 ng/mL, and presence of macrovessel invasion were used to dichotomize for univariate analyses. The Child Pugh score and etiology of liver disease were also examined. A *p* value of < 0.05 was considered statistically-significant under log-rank tests. No factor showed significance in univariate analysis though analyses were limited due to small small sample sizes. Key: CI = confidence interval. ETOH = alcohol. NAFLD = non-alcoholic fatty liver disease. I = sample size insufficient for analysis.

### CTC isolation by IE/FACS

Five patients in the HCC cohort showed greater than 10 CTC per 7.5 mL detected by CellSearch. CTCs were then isolated via IE/FACS performed on the remaining blood samples collected from these patients. IE/FACS was also performed on the specimen from Hep 25, the patient removed from the NMLD cohort for the finding of a liver mass with portal vein thrombosis. Absolute CTC counts by CellSearch and IE/FACS for these samples are provided in Additional file 1.

### CTC, PBMC, and FFPE sequencing performance

Sequencing of adequate DNA samples from CTCs, FFPE tumor samples, and banked PBMC from the study cohort (Figure 1, Table 3) was performed. Paired FFPE tumor and/or PBMC from patients with adequate CTC DNA for sequencing were available in two cases; two additional cases with paired FFPE tumor and PBMC samples available without adequate CTC DNA also were analyzed from the HCC cohort (Figure 1). Sequencing performance according to sample type is displayed in Table 3. Sequencing performance data for FFPE tumor samples and banked PBMC (both a source of DNA not requiring WGA) were combined due to small sample sizes, for comparison to WGA DNA from CTCs (Table 3). The mean amplicon read depth was lower (2258 versus 2954, *p* < 0.01) and proportion of targeted bases with sequencing coverage of ≥ 100x was significantly lower in CTC samples (43%) than in FFPE tumor plus PBMC samples (87%) (*p* < 0.025), using two-tailed t-tests. The mean number of variant calls per sample was higher in CTC samples compared to FFPE samples (9 vs. 2, *p* < 0.04), though the mean frequency of individual variant alleles was significantly lower (36% vs. 60%, *p* < 0.001) (two-tailed t-tests). Reproducibility of sequencing results was demonstrated by 3 samples run in duplicate (data not shown).

**Table 3:** **Sequencing performance by sample type**

Sample type	CTC WGA DNA (*n* = 5)	FFPE Tumor DNA (*n* = 6) and PBMC DNA (*n* = 3) (*n* = 9 total^a^)	*p*value (two-tailed t-test)
Mean read length	74 bp	76 bp	NS
Mean mapped reads per sample	653,878 bp	668,633 bp	NS
Mean amplicon read depth (std. dev)	2258 (4389)	2954 (1379)	*p* < 0.01
Proportion with coverage > 20x	50%	97%	*p* < 0.0002
Proportion with coverage > 100x	43%	88%	*p* < 0.026
Mean non-synonymous variant calls per sample	9	2^b^	*p* < 0.03
Mean variant allele frequency	37%	61%^b^	*p* < 0.0001

^a^Data from FFPE and PBMC DNA samples were combined for sequencing performance analyses (but not for genotype analyses) due to small sample size and similar observed coverage. NS = not significant. ^b^PBMC samples (germline DNA) were excluded from variant analyses, *n* = 3.

### Sequencing results: variants, SNPs and mutation calls

**Figure 4:**
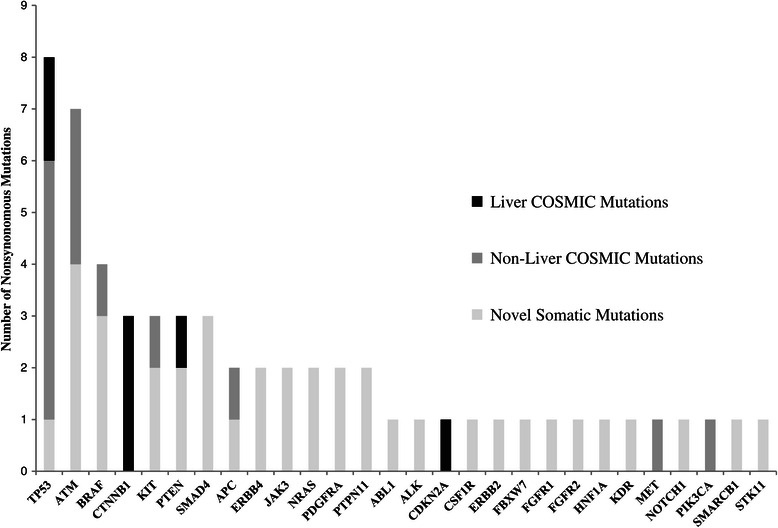
**Summary of somatic, non-synonymous mutations.** Occurring in CTC WGA DNA (n = 5) and/or tumor DNA (n = 6).

Eighty-six variants overall, 58 of which were predicted to be protein-altering, were identified from all of the CTC and FFPE tumor samples combined. Approximately 54% were low-frequency (occurring in less than 10% of the individual sample), among which 93% were from CTC-derived DNA. Fifty-eight somatic, non-synonymous variants were called mutations if a matching mutation has been described in liver cancer, if the variant shared the same amino acid residue as a COSMIC mutation in any cancer type, and/or if the variant allele frequency was greater than 5% but the variant was not a known SNP and not present in any PBMC sample [28]. Frameshift mutations were excluded from analysis due to known limitations of ion semiconductor sequencing to accurately detect frameshift mutations. Characteristic mutations in HCC (*TP53*, *PTEN*) were identified in CTC-derived DNA from two cases. Figure 4 displays a summary of the somatic, non-synonymous mutations identified in CTC and FFPE tumor samples combined. A listing of all somatic, non-synonymous mutations (excluding frameshift) detected according to sample type is provided in Additional file 2. In one HCC case with matched CTC, FFPE tumor, and PBMC DNA, 8 SNPs were present and concordant in both FFPE tumor and PBMC DNA; 5 of these (63%) were detected in the CTC DNA. Neither was identified in the paired CTC DNA.

## Discussion

The ability to detect and characterize malignant cells in circulation holds enormous promise as a biomarker in HCC, a grim cancer challenged by the inability of conventional noninvasive diagnostic and staging modalities to encompass its great clinical and biological heterogeneity, as well as by a scarcity of tumor tissue available for diagnostic or research purposes. In this study, at least one CTC was detected in 8/20 (40%) of patients with metastatic HCC, compared to 1/9 (11%) of eligible NMLD patients using the CellSearch System. Though the cut-point of ≥ 1 CTC/7.5 mL did not achieve significance between the two groups, a cut-point of ≥ 2 CTCs/7.5 mL was significant, positive in 7/20 (35%) HCC patients compared with none in the NMLD cohort (*p* = 0.04), consistent with prior reports [14,15]. The one eligible NMLD control patient with CTC count of 1/7.5 mL was subsequently found to have ultrasound findings suggestive of underlying tumor, although no formal HCC diagnosis was made, and thus he was not removed from the control cohort. Our findings confirm the limited existing data suggesting that circulating EpCAM-positive epithelial cells are rare in patients with non-malignant liver diseases, and that EpCAM-positive cells in HCC patients are generally of tumor origin [14].

Corroborating the prognostic value of EpCAM-positive CTCs in other recent series [14,15], the detection of CTCs in the HCC cohort of this study was significantly associated with high AFP and the presence of vascular invasion, and there was a non-significant trend toward poorer overall survival in patients with detectable CTCs. These findings support the value of CTCs as a prognostic biomarker in metastatic HCC and suggest future potential roles for CTCs in treatment decision-making as well as for stratification in clinical research, which historically has been challenged by the great prognostic heterogeneity of this disease [29].

The unexpected finding of high CTC levels in a patient initially enrolled to the NMLD cohort, who subsequently was removed for ineligibility due to the finding of a new liver mass with vascular invasion on ultrasound suggestive of HCC, raises the intriguing possibility that CTC detection also may be associated with vascular invasion and poor prognosis in earlier stages of disease. This incidental finding, along with recent results of Schulze *et al.* and Sun *et al.* indicating prognostic value of CTC detection in patients with localized HCC [14,15], suggest an important potential role for CTCs as a biomarker of occult vascular invasion, recurrence risk, and overall survival in patients with apparent localized disease undergoing evaluation for surgery or transplantation.

Our finding that EpCAM-positive CTCs are associated with high AFP and the presence of vascular invasion is in keeping with the results of others [14,15] which indicate that EpCAM-positive CTCs have biologic relevance as a diagnostic and prognostic biomarker in HCC. EpCAM expression and an EpCAM-positive gene expression signature are associated with poor differentiation, high AFP levels, and activation of Wnt-β-catenin signaling pathways [30-32]. EpCAM-positive HCC cells also express markers associated with cancer stem cells and the epithelial to mesenchymal transition, supporting a hypothesis that EpCAM enrichment identifies stem-like cells with potential for metastasis [15,30,31,33].

A key unanswered question is whether EpCAM is the optimal marker for CTC enrichment in HCC. Unlike other epithelial tumor types which demonstrate nearly universal EpCAM expression [34], EpCAM is not expressed on mature hepatocytes and is expressed in only approximately 35% to 60% of HCC tumors by immunohistochemistry or PCR-based methods [30,31,35-37]. Thus, it is possible that non-EpCAM-expressing HCC cells exist in circulation and are undetectable by technologies employing EpCAM enrichment, which may account for our inability to detect CTCs in some of our HCC patients. Small series of non-EpCAM-based CTC isolation methods, such selection for the expression of asialoglycoprotein receptor or pancytokeratin or by cell size, suggest numerically higher incidence of detectable CTCs in metastatic HCC patients than has been reported with CellSearch, though the data are limited by small sample sizes and are not comparative [16,17,38]. Optimal CTC isolation and enrichment in HCC may require combining EpCAM with other markers.

Beyond using CTC detection and enumeration as a prognostic biomarker, however, CTCs offer a dynamic window into the evolution of metastatic disease. The advent of next-generation sequencing has revealed a remarkable degree of heterogeneity within individual tumors and between primary tumors and their metastases [39]. With increasingly sensitive and precise technologies for the detection and molecular profiling of rare cells, the genomic interrogation of CTCs may offer a powerful new tool to characterize, and someday to target, the dominant tumor subclones responsible for treatment resistance or metastatic progression. Heitzer *et al.* recently reported the first comprehensive genomic profiling of single CTCs using array comparative genomic hybridization and next-generation sequencing in a study of 37 patients with metastatic colorectal cancer [18]. Among the 6 patients with adequate (>10) CTCs isolated for genomic profiling, concordance on copy number changes and characteristic driver mutations including *PIK3CA*, *APC*, and *KRAS* was shown, along with many additional mutations in the CTCs which were later found to be present at subclonal levels in the primary tumors by deep sequencing. Interestingly, heterogeneity was observed between CTCs isolated from the same patient at the same time-point.

This pilot study represents the first report of efficient isolation and next-generation sequencing of CTCs in HCC, to our knowledge. In this study, ion semiconductor next-generation sequencing showed a significantly higher proportion of targeted bases with at least 100x coverage depth among FFPE tumor and PBMC samples (87%) compared to CTC-derived DNA samples (43%) (*p* < 0.025). The disparate coverage depths according to sample type may be due in part to the use of an adaptor-ligation PCR WGA method which has been associated with allelic loss; alternate methods of amplification such as multiple displacement may mitigate this effect [40-42]. An alternate or contributory factor leading to the difference in allele frequency between sample types, as well as to the mutational disagreements between FFPE and CTC samples, may be the inherent heterogeneity of individual CTCs which were pooled for WGA from each patient [43]. WGA may also introduce low frequency variants by artifact [40,41].

In our study, 86 variants were identified from CTC and FFPE tumor samples. One half of the variants were low frequency (<10%) and derived predominantly from the CTC DNA samples. While again this finding could be due to coverage bias or artifact arising from WGA, these results are also consistent with the findings from Heitzer *et al.* in a colorectal cancer cohort [18], which suggest significant inter-CTC heterogeneity and could explain the prevalence of low-frequency variants arising from pooled DNA derived from multiple CTCs from an individual patient. Characteristic mutations associated with HCC (including *TP53* and *PTEN*) were identified in CTC-derived DNA, consistent with tumor origin [44]. The overall sequencing accuracy in this study was demonstrated by several cases with available paired PBMC, CTC, and tumor DNA samples showing concordance on SNP calls, along with reproducibility of results in duplicate runs. A significant limitation of the exploratory sequencing in this pilot study, however, was its small sample size, along with the limited proportion of cases with paired CTC, FFPE tumor, and PBMC DNA available.

## Conclusions

This study strongly supports that circulating epithelial cells are detectable in HCC patients, including via the CellSearch assay; and that these cells are EpCAM-positive tumor cells in circulation, rather than benign epithelial cells released in the setting of liver injury. These findings are based on significant CTC detection in HCC but not in NMLD cohorts, associations between CTC detection and HCC prognostic markers, and the demonstration of characteristic HCC mutations in DNA derived from purified CTCs. The significant association with macrovessel invasion and elevated AFP in this study, along with a trend towards poorer survival, indicate the potential value of CTC detection as a prognostic biomarker in metastatic HCC. Prospective analyses of CTCs in earlier stages of disease are warranted to determine surrogacy for vascular invasion in patients undergoing evaluation for surgery or liver transplantation. In parallel, we demonstrate that CTCs offer a source of non-invasive tumor DNA for next-generation sequencing and molecular profiling efforts in HCC. Future studies to determine the optimal CTC isolation technology, cut-points by assay and population, and methods for single-cell CTC molecular characterization are essential to develop CTCs as a clinical biomarker as well as a research tool in this grim, complex disease in urgent need of new biomarkers and therapeutic targets.

## Additional files

Additional file 1:
**Absolute CTC counts by CellSearch and IE/FACS.**

Additional file 2: **List of somatic, non-synonymous mutations identified by targeted sequencing.** Key: VAF = variant allele frequency. SNV = single nucleotide variant. Met = metastasis. * = Same amino acid residue as a liver COSMIC mutation. Variants were identified as somatic mutations if non-synonymous and: a matching COSMIC^27^ mutation has been described in liver cancer (highlighted in yellow), the variant shared the same amino acid residue as a COSMIC mutation, and/or if the variant allele frequency was greater than 5% but the variant was not a known SNP and not present in any PBMC sample tested. Frameshift mutations were excluded due to known limitations of ion semiconductor sequencing on frameshift calls.
