# Supplementary material for: Circulating tumor cells in hepatocellular carcinoma: a pilot study of detection, enumeration, and next-generation sequencing in cases and controls
Source: BMC Cancer. 2015 Mar 31;15:206. doi: 10.1186/s12885-015-1195-z (PMC4399150; doi:10.1186/s12885-015-1195-z)
Supplement: Additional file 1: — Absolute CTC counts by CellSearch and IE/FACS. [file 12885_2015_1195_MOESM1_ESM.docx]

| Additional file 1. Absolute CTC counts by CellSearch and IE/FACS | | |
| --- | --- | --- |
| Patient ID | CellSearch CTC/mL | IE/FACS CTC/mL |
| Hep 2 | 2.3 | 2.4 |
| Hep 3 | 2 | 0.7 |
| Hep 5 | 2.4 | 1.6 |
| Hep 10 | 5.6 | 0.5 |
| Hep 18 | 10.3 | 1.0 |
| Hep 25^a^ | 2.7 | 0.9 |
| ^a^NMLD control patient. | | |
