# Supplementary material for: Circulating tumor cells in hepatocellular carcinoma: a pilot study of detection, enumeration, and next-generation sequencing in cases and controls
Source: BMC Cancer. 2015 Mar 31;15:206. doi: 10.1186/s12885-015-1195-z (PMC4399150; doi:10.1186/s12885-015-1195-z)
Supplement: Additional file 2: — List of somatic, non-synonymous mutations identified by targeted sequencing. Key: VAF = variant allele frequency. SNV = single nucleotide variant. Met = metastasis. * = Same amino acid residue as a liver COSMIC mutation. Variants were identified as somatic mutations if non-synonymous and: a matching COSMIC27 mutation has been described in liver cancer (highlighted in yellow), the variant shared the same amino acid residue as a COSMIC mutation, and/or if the variant allele frequency was greater than 5% but the variant was not a known SNP and not present in any PBMC sample tested. Frameshift mutations were excluded due to known limitations of ion semiconductor sequencing on frameshift calls. [file 12885_2015_1195_MOESM2_ESM.docx]

Additional file 2. List of somatic, non-synonymous mutations identified by targeted sequencing

| Sample | Tissue | Gene | Chromosome | Genomic Position | Coverage Depth | VAF  (%) | Amino Acid Change | Mutation Type | Present in COSMIC Liver^27^ |
| --- | --- | --- | --- | --- | --- | --- | --- | --- | --- |
| Hep 2 | CTC | ALK | 2 | 29432709 | 1296 | 98.4 | P1260L | nonsynonymous SNV | No |
| Hep 2 | CTC | ATM | 11 | 108236054 | 43 | 7.0 | D2997G | nonsynonymous SNV | No |
| Hep 2 | CTC | BRAF | 7 | 140481475 | 2814 | 8.5 | D445N | nonsynonymous SNV | No |
| Hep 2 | CTC | JAK3 | 19 | 17945689 | 87 | 5.7 | M724T | nonsynonymous SNV | No |
| Hep 2 | CTC | KDR | 4 | 55955116 | 4036 | 6.5 | W1143X | stopgain SNV | No |
| Hep 2 | CTC | KIT | 4 | 55597499 | 823 | 5.6 | D716G | nonsynonymous SNV | No |
| Hep 2 | CTC | MET | 7 | 116339641 | 59 | 5.1 | E168G | nonsynonymous SNV | No |
| Hep 2 | CTC | NOTCH1 | 9 | 139397733 | 56 | 7.1 | S1690P | nonsynonymous SNV | No |
| Hep 2 | CTC | PDGFRA | 4 | 55152039 | 1901 | 6.2 | V824A | nonsynonymous SNV | No |
| Hep 2 | CTC | PTPN11 | 12 | 112926840 | 747 | 46.6 | D487V | nonsynonymous SNV | No |
| Hep 2 | CTC | STK11 | 19 | 1220452 | 189 | 18.5 | L182P | nonsynonymous SNV | No |
| Hep 3 | CTC | ABL1 | 9 | 133738315 | 34 | 8.8 | R239C | nonsynonymous SNV | No |
| Hep 3 | CTC | ATM | 11 | 108180957 | 3149 | 14.4 | A1945T | nonsynonymous SNV | No |
| Hep 3 | CTC | ATM | 11 | 108225600 | 3176 | 5.4 | E2950G | nonsynonymous SNV | No |
| Hep 3 | CTC | CSF1R | 5 | 149452997 | 7106 | 5.9 | E317K | nonsynonymous SNV | No |
| Hep 3 | CTC | FGFR2 | 10 | 123274812 | 1134 | 6.5 | I280T | nonsynonymous SNV | No |
| Hep 3 | CTC | PTEN | 10 | 89685272 | 240 | 6.7 | F56S | nonsynonymous SNV | No |
| Hep 3 | CTC | PTEN | 10 | 89711893 | 1239 | 5.1 | Q171X | stopgain SNV | COSM5149 |
| Hep 3 | CTC | SMAD4 | 18 | 48604752 | 5696 | 16.8 | I525T | nonsynonymous SNV | No |
| Hep 3 | CTC | SMARCB1 | 22 | 24133992 | 1737 | 99.2 | P48R | nonsynonymous SNV | No |
| Hep 3 | CTC | TP53 | 17 | 7579875 | 204 | 6.9 | P13L | nonsynonymous SNV | No |
| Hep 3 | Met | TP53 | 17 | 7577121 | 5756 | 85.1 | R114C* | nonsynonymous SNV | COSM10659* |
| Hep 5 | Met | PIK3CA | 3 | 178952058 | 30 | 16.7 | Y1038C | nonsynonymous SNV | No |
| Hep 5 | Met | TP53 | 17 | 7577121 | 33 | 75.8 | R114C* | nonsynonymous SNV | COSM10659* |
| Hep 5 | Met | TP53 | 17 | 7578191 | 119 | 5.9 | Y61H | nonsynonymous SNV | No |
| Hep 5 | Met | TP53 | 17 | 7578257 | 118 | 11.0 | E39K | nonsynonymous SNV | No |
| Hep 5 | Met | TP53 | 17 | 7578404 | 2526 | 7.9 | C17R | nonsynonymous SNV | No |
| Hep 8 | Met | CTNNB1 | 3 | 41266100 | 4886 | 21.4 | S33A | nonsynonymous SNV | COSM5683 |
| Hep 8 | Met | ERBB4 | 2 | 212578310 | 1631 | 31.3 | E316V | nonsynonymous SNV | No |
| Hep 10 | CTC | APC | 5 | 112175942 | 1452 | 7.2 | K1551E | nonsynonymous SNV | No |
| Hep 10 | CTC | ATM | 11 | 108205808 | 8037 | 30.3 | D2708G | nonsynonymous SNV | No |
| Hep 10 | CTC | ATM | 11 | 108236071 | 8021 | 7.0 | N3003D | nonsynonymous SNV | No |
| Hep 10 | CTC | BRAF | 7 | 140481400 | 6308 | 5.8 | T470A | nonsynonymous SNV | No |
| Hep 10 | CTC | FBXW7 | 4 | 153247309 | 6901 | 5.8 | M380T | nonsynonymous SNV | No |
| Hep 10 | CTC | JAK3 | 19 | 17947995 | 1656 | 5.4 | S577G | nonsynonymous SNV | No |
| Hep 10 | CTC | NRAS | 1 | 115256505 | 237 | 7.6 | D69V | nonsynonymous SNV | No |
| Hep 10 | CTC | PTEN | 10 | 89685304 | 1803 | 6.8 | I67V | nonsynonymous SNV | No |
| Hep 10 | CTC | TP53 | 17 | 7577025 | 1384 | 7.0 | K146E | nonsynonymous SNV | No |
| Hep 10 | Primary | CTNNB1 | 3 | 41266097 | 6283 | 10.0 | D32Y | nonsynonymous SNV | COSM5661 |
| Hep 10 | Primary | TP53 | 17 | 7578205 | 3756 | 37.1 | S56I | nonsynonymous SNV | No |
| Hep 11 | Met | CDKN2A | 9 | 21971186 | 1605 | 54.1 | R58X | stopgain SNV | COSM12473 |
| Hep 11 | Met | CTNNB1 | 3 | 41266124 | 3548 | 46.6 | T41A | nonsynonymous SNV | COSM5664 |
| Hep 11 | Met | HNF1A | 12 | 121431410 | 3936 | 46.8 | K205T | nonsynonymous SNV | No |
| Hep 18 | CTC | APC | 5 | 112175222 | 1078 | 7.9 | I1311V | nonsynonymous SNV | No |
| Hep 18 | CTC | ATM | 11 | 108173641 | 2525 | 5.3 | L1794Q | nonsynonymous SNV | No |
| Hep 18 | CTC | BRAF | 7 | 140481400 | 88 | 9.1 | T470A | nonsynonymous SNV | No |
| Hep 18 | CTC | BRAF | 7 | 140481438 | 92 | 7.6 | I457T | nonsynonymous SNV | No |
| Hep 18 | CTC | ERBB2 | 17 | 37880229 | 682 | 9.7 | N728T | nonsynonymous SNV | No |
| Hep 18 | CTC | ERBB4 | 2 | 212589823 | 8042 | 29.9 | G240E | nonsynonymous SNV | No |
| Hep 18 | CTC | FGFR1 | 8 | 38282163 | 2617 | 6.6 | V259A | nonsynonymous SNV | No |
| Hep 18 | CTC | KIT | 4 | 55593470 | 3782 | 9.8 | L543M | nonsynonymous SNV | No |
| Hep 18 | CTC | KIT | 4 | 55597526 | 68 | 5.9 | K725R | nonsynonymous SNV | No |
| Hep 18 | CTC | NRAS | 1 | 115258742 | 3104 | 9.5 | V14L | nonsynonymous SNV | No |
| Hep 18 | CTC | PTPN11 | 12 | 112926843 | 2343 | 99.9 | I488T | nonsynonymous SNV | No |
| Hep 18 | CTC | SMAD4 | 18 | 48593436 | 83 | 8.4 | D396G | nonsynonymous SNV | No |
| Hep 19 | Primary | ATM | 11 | 108180932 | 498 | 5.4 | L1936F | nonsynonymous SNV | No |
| Hep 25 | CTC | SMAD4 | 18 | 48604727 | 7988 | 99.9 | S517G | nonsynonymous SNV | No |

Key: VAF=variant allele frequency. SNV=single nucleotide variant. Met=metastasis. *=Same amino acid residue as a liver COSMIC mutation. Variants were identified as somatic mutations if non-synonymous and: a matching COSMIC^27^ mutation has been described in liver cancer (highlighted in yellow), the variant shared the same amino acid residue as a COSMIC mutation, and/or if the variant allele frequency was greater than 5% but the variant was not a known SNP and not present in any PBMC sample tested. Frameshift mutations were excluded due to known limitations of ion semiconductor sequencing on frameshift calls.
